# Impact of diagnostic bone biopsies on the management of non-vertebral osteomyelitis: A retrospective cohort study

**DOI:** 10.1097/MD.0000000000016954

**Published:** 2019-08-23

**Authors:** Cole B. Hirschfeld, Shashi N. Kapadia, Joanna Bryan, Deanna P. Jannat-Khah, Benjamin May, Ole Vielemeyer, Ernie L. Esquivel

**Affiliations:** a Weill Cornell Medical College; b Department of Medicine, Columbia University College of Physicians and Surgeons; c Division of Infectious Diseases; d Division of General Internal Medicine, Weill Department of Medicine; e Division of Interventional Radiology, Department of Radiology, Weill Cornell Medicine, New York, NY, USA.

**Keywords:** diagnostic decision making, osteomyelitis, procedures

## Abstract

Optimal antibiotic management of patients with osteomyelitis remains a challenge for many clinicians. Although image-guided bone biopsy (IGB) remains the gold standard, its role in confirming diagnosis and guiding antibiotic management is not clear in patients with non-vertebral osteomyelitis.

To determine the diagnostic yield of IGB and its impact on antibiotic management in non-vertebral osteomyelitis.

Retrospective cohort study.

Urban academic medical center.

Patients admitted for non-vertebral osteomyelitis who underwent image-guided bone biopsy.

Primary outcomes were microbiologic and histopathological results. We evaluated the impact of IGB on clinician-initiated changes in antibiotic regimen before and after biopsy.

We evaluated 203 bone biopsies in 185 patients with clinical suspicion of osteomyelitis. 79% of patient received antibiotics prior to biopsy. Bone cultures were positive in 28% and histopathology confirmed osteomyelitis in 29%, but concordance was poor. Furthermore, clinical suspicion of infection was much higher, given that 68% received empiric antibiotics. Leukocytosis was significantly associated with positive cultures in multivariate analysis. There was no statistically significant correlation between antibiotic management and bone culture results. When culture yielded an organism, empiric regimens were kept the same, broadened or narrowed with equal frequency; targeted regimens were chosen only in 4 cases. Despite negative cultures in 98/138 cases having received empiric treatment, antibiotics were discontinued in only 8 cases. Even when empiric treatment was not given, negative cultures did not dissuade clinicians from eventual antibiotic use in a significant number of cases (17/48). In 46/71 patients whose final regimen included vancomycin, there was no evidence of current or past infection with MRSA.

In patients with non-vertebral osteomyelitis, the diagnostic yield of image-guided bone biopsy is low, and clinicians frequently make decisions regarding antibiotic management that are not aligned with culture results.

## Abstract 2

Supplemental Digital Content is available in the text

## 1 Introduction

Osteomyelitis is a destructive inflammatory bone disease due to bacterial or fungal infection. The underlying pathophysiology involves contiguous spread of infection, or hematogenous seeding.^[1]^ The incidence of osteomyelitis may be increasing due to improved sensitivity of diagnostic imaging or rising prevalence of risk factors, such as diabetes.^[2,3]^ It incurs significant morbidity and substantial health care costs, and requires a multidisciplinary treatment approach.

Bone biopsies are commonly used for diagnosis, but their outcomes can vary greatly. Prior studies have found that the yield is higher when more material is obtained, aspirate appears purulent, and antibiotics had been withheld and in hematogenous osteomyelitis (90%).^[4–6]^ Clinical guidelines recommend tissue sampling for suspected vertebral osteomyelitis.^[4]^ In contrast, biopsies in diabetic foot osteomyelitis (usually due to contiguous spread of infection) are not routinely recommended.^[4]^ Experts advocate biopsy only when there is diagnostic uncertainty, inadequate wound culture data or empiric therapy failure.^[7,8]^ For all types of non-vertebral osteomyelitis the diagnostic yield is lower (34–68%).^[6,9,10]^ In diabetic foot infections, where much of the literature is focused, a combination of clinical and radiologic findings may establish the diagnosis with greater certainty than biopsy and antibiotics are often given even with negative microbiology.^[11–15]^ The clinical utility of diagnostic bone biopsies in this subgroup is less certain and only limited data are available.^[6,9,10]^ A small, retrospective study concluded that treatment achieved a higher rate of remission when specifically targeting the identified bone organisms.^[16]^

The rapidly expanding field of interventional radiology has made image-guided biopsies (IGB) more readily available. Our aim was to determine the diagnostic yield of IGB and the factors predicting a positive result and we asked how frequently the procedure resulted in a change in antibiotic management in patient with non-vertebral osteomyelitis.

## 2 Methods

### 2.1 Study population

We retrospectively reviewed all cases of fluoroscopy- or CT-guided biopsy (subsequently designed IGB) performed on patients with non-vertebral osteomyelitis between 2009 and 2016 at New York-Presbyterian Hospital/Weill Cornell Medical Center. Non-vertebral osteomyelitis was defined as suspected or confirmed infection of any bone excluding, the cervical, thoracic, and lumbar spine. Subjects who had undergone sacral or coccygeal biopsy were included when an overlying decubitus ulcer was documented. Biopsy procedures were conducted by Interventional Radiology in accordance with standard practice at our institution. CT or fluoroscopic guidance was used to target the region deemed most suspicious on imaging. In cases where an ulcer was present, a needle path excluding the ulcer was chosen to minimize the chance of microbial contamination. Core samples were taken when possible and divided between surgical pathology and microbiology. When core samples were not possible, aspirates were obtained for culture. Patients who underwent open (surgical) bone biopsies, were under 18 years of age, died within five days of biopsy or had insufficient follow-up data were excluded.

This study was approved by the institutional review board and a waiver of informed consent was obtained.

### 2.2 Biopsy yield

Microbiology-positive cases were defined as bone biopsies in which an organism grew from culture (including enrichment broth) or was seen on direct staining. Bone histology was considered positive if acute or chronic osteomyelitis was identified. Acute osteomyelitis was defined by the presence of acute inflammatory cells and/or osteonecrosis. Chronic osteomyelitis was defined as the presence of marrow fibrosis and devitalized or remodeled bone with or without lymphocytes or plasma cells.^[17,18]^ Because of the heterogeneity of phrases used in the original pathology reports (leading to some ambiguity), a pathologist specializing in bone pathology was consulted to clarify ambiguous reports.

### 2.3 Impact on antibiotic management

Charts were reviewed by three physicians (an Infectious Disease fellow [SNK] and attending physician [OV], a hospitalist attending [ELE]) and a medical student (CBH). *“Empiric antibiotics”* were defined as antibiotics initiated after biopsy, prior to culture results being available. *“Final antibiotics”* referred to antibiotics chosen after culture data from biopsy were available. The post-biopsy period was chosen to represent empiric therapy due to the large number of cases in which antibiotics were held prior to biopsy with the presumed goal of increasing culture yield. If the empiric and final antibiotic regimen were the same, this was designated as “*No change*.” The impact on antibiotic management was categorized as initiated, discontinued, broadened, narrowed, or targeted (Supplementary Table 1). Antibiotics used concurrently to treat other infections, e.g. urinary tract infections or pneumonia, were factored into the calculation of the antibiotic-free period but were included in “empiric” and “final regimen” only if dual indication was specifically documented in the provider's note.

### 2.4 Statistical analysis

Differences in frequency distribution were compared using the χ^2^, Wilcoxon rank-sum and Fisher's exact tests. Differences in continuous variables were compared using analysis of variance. Due to the commonness of positive bone cultures, relative risk ratios were calculated to determine the effect of clinical and procedural characteristics on the outcome of bone culture yield using Poisson regression with robust standard errors. The following factors were analyzed in bivariate and fully adjusted models to determine if they had a significant effect on the bone culture yield: age, sex, anatomic location of the biopsy, biopsy needle size, number of biopsies cultured in the microbiology lab, white blood cell (WBC) count, presence of overlying cellulitis, presence of an overlying ulcer, antibiotic-free days prior to biopsy, and comorbidities including diabetes, end-stage renal disease, paraplegia, peripheral neuropathy, peripheral vascular disease, and prior amputations. Erythrocyte sedimentation rate, C-reactive protein, blood cultures, and wound cultures were excluded from the multivariate analysis due to insufficient data across all study patients. Fever was excluded because only one patient was recorded with temperature ≥ 38.0°C within 48 h of the biopsy. The relationship between each individual characteristic and bone culture yield was investigated both individually and in a full model adjusting for all other characteristics. Statistical testing was performed using Stata version 14.

## 3 Results

Between 2009 and 2016, 212 IGBs were performed for suspected non-vertebral osteomyelitis. Nine were excluded due to inadequate follow-up. We ultimately analyzed 203 biopsies performed in 185 patients. Biopsies were performed in the inpatient setting in 195 cases, of whom 155 (79%) had been admitted with a primary diagnosis of osteomyelitis. Osteomyelitis was diagnosed radiologically in all patients, most commonly by MRI. Several patients underwent more than one imaging modality. Nearly half were patients with diabetes and close to a quarter had peripheral vascular disease. A single bone was biopsied in over 90% of patients, most commonly those of the foot (61%). An ulcer overlay the site of suspected osteomyelitis in 75% of cases and cellulitis was present in 38%. Fourteen patients had one or more repeat biopsies of the same bone. In most cases (160/203, 79%), antibiotics were given within 30 days prior to the biopsy, and 61 (30%) patients received antibiotics prior to admission. The median number of days off antibiotics prior to biopsy was two days (IQR 0, 10) (Table 1).

**Table 1:**
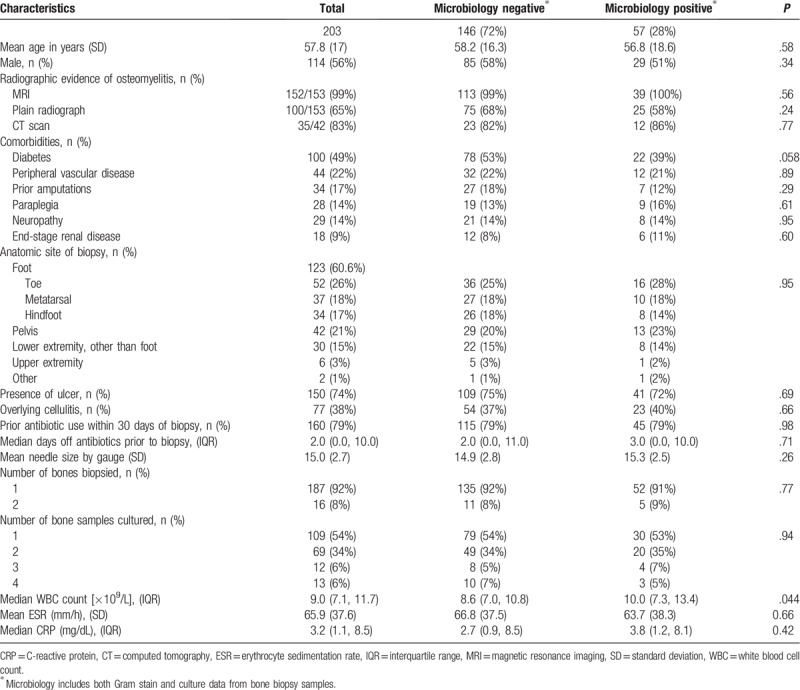
Demographics and characteristics of bone biopsy cases.

### 3.1 Biopsy yield

Samples for histopathological analysis were sent in 115 cases (57%). Biopsies were conclusive in 51%: osteomyelitis was seen in 33 cases (29%) and negative in 26 cases (22%). Biopsies were inconclusive in 56 cases (49%). One case yielded an alternative diagnosis of diffuse large B-cell lymphoma.

Bone samples were sent for microbiology in all 203 cases. A positive result was obtained in 57 (28%), including six with a positive Gram stain only. The most commonly isolated organisms were *Staphylococcus aureus*, *Streptococcus* species and Enterobacteriaceae (Table 2). Within the subset of 115 cases for which histopathology was available, positive concordance was seen in only 8 (7%). Two or more organisms (polymicrobial) were isolated in 13 cases.

**Table 2:**
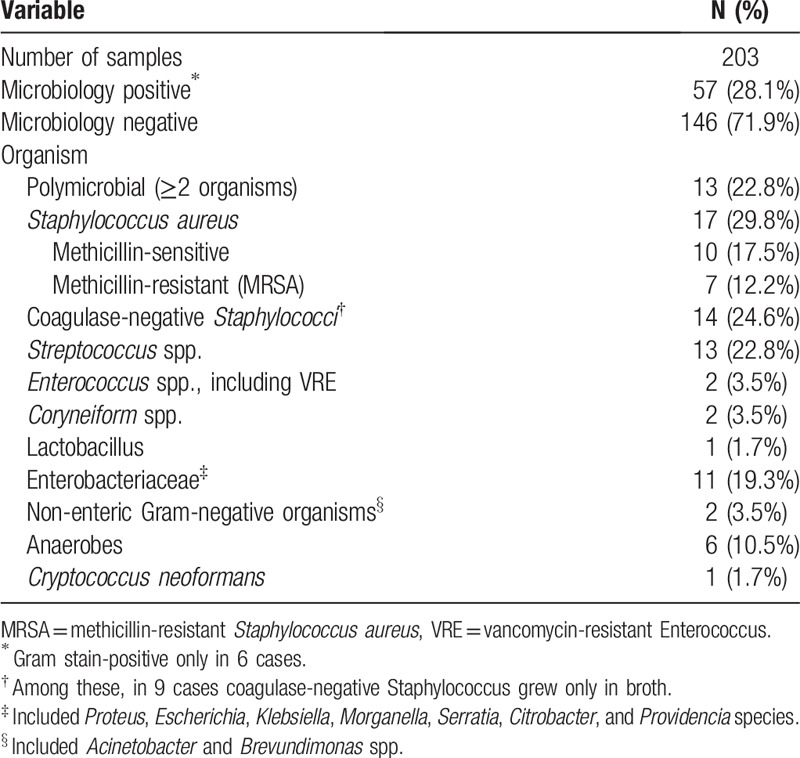
Microbiology results from bone biopsy.

Superficial wound cultures were performed in 60 cases, 49 (81.6%) of which were positive, often polymicrobial (29 cases). *S aureus* was seen most commonly, and in 10% *Pseudomonas* was isolated (Supplemental Table 2). More than half of the wound culture-positive samples (29 cases) had negative bone cultures. Of the 20 samples where bone and wound cultures were both positive (41%), only 4 had fully concordant results and in an additional two cases, at least one organism was seen from each sample.

We then looked to see whether we could identify factors affecting the microbiological yield. There was no correlation between positive cultures and the number of samples cultured, biopsy needle size, prior antibiotic use, number of antibiotic-free days, or comorbid conditions. Diabetes was associated with fewer culture-positive biopsies, but this did not reach statistical significance (RR = 0.63, 95% CI 0.37, 1.07; *P* = .088). Only an elevated WBC count was predictive of a positive microbiology results (RR 1.05, 95% CI 1.01, 1.10; *P* = .02) (Table 3).

**Table 3:**
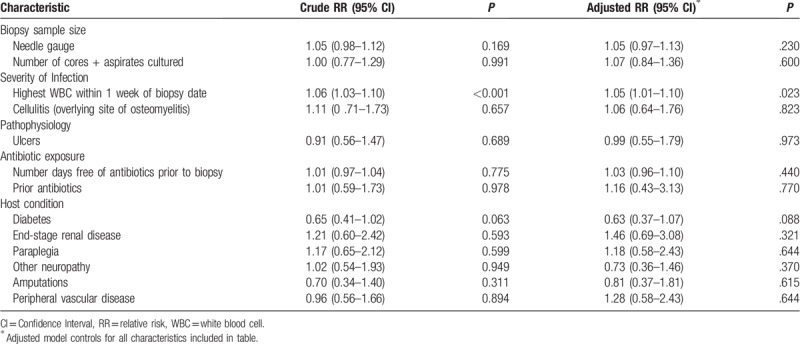
Predictors of positive microbiology result after bone biopsy.

### 3.2 Impact on antibiotic management

Finally, we looked at the clinical utility of IGB by assessing whether it led to a change in antibiotic management after finalized biopsy results (Table 4). Culture results revealed possible inadequate antibiotic coverage in only 3 cases. Empiric antibiotics were administered in 138 (68%) cases. Among these, 40 (29%) were culture-positive. Nearly half of patients remained on the same antibiotic regimen, whereas antibiotics were either broadened, narrowed, or targeted in 21 (53%). Cultures were negative in 98 (71%) patients who had received empiric antibiotics, yet antibiotics were continued unchanged (52 cases) or broadened (9 cases). In 29 (30%) patients, antibiotics were narrowed; however, antibiotics were discontinued only in 8 (8%) patients. Empiric antibiotics were not administered post-biopsy in 65 patients. Of these, 17 (26%) had positive microbiology and in 13 patients, antibiotics were initiated, 4 of which were targeted to a specific pathogen. Despite negative cultures in 48 cases, antibiotics were, nonetheless, initiated in 17 (35%) patients. In summary, although the overall microbiology yield was 28%, final antibiotics were, nevertheless, prescribed in 79% (160/203).

**Table 4:**
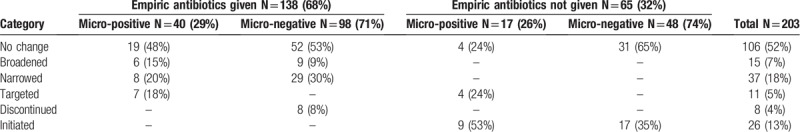
Changes in antibiotic spectrum from empiric to final therapy.

More than two-thirds of all empiric antibiotic regimen included vancomycin (95/138 cases). Almost all final regimens were broad-spectrum and often included intravenous medication, with 71 (44%) cases receiving vancomycin. However, in 46/71 (65%) vancomycin was given even though no current or past infection with MRSA could be identified. Few patients had evidence of methicillin-resistant *S aureus* (MRSA) infection: 7 grew it in bone, 5 in wound culture; a further 32 had a history of MRSA. This left 159 cases with neither current nor prior MRSA infection/colonization. Nonetheless, 55 of them were discharged with a parenteral anti-MRSA antibiotic.

## 4 Discussion

Optimal management of patients with osteomyelitis is a challenge for clinicians particularly since cure requires prolonged courses of antibiotics.^[8]^ Although bone biopsy remains a gold standard for non-vertebral osteomyelitis, clinicians frequently rely on clinical information and radiologic studies to establish the diagnosis and biopsies are more commonly performed to guide antibiotic choices.^[19]^ In this large retrospective study conducted in an academic hospital we looked at the impact of IGBs on management of clinically suspected non-vertebral osteomyelitis, focusing on its diagnostic yield and its clinical utility by reviewing the impact on the antimicrobial therapy.

The overall microbiologic yield was low at 28%. Furthermore, we found poor concordances between bone and wound cultures and between bone cultures and histology. The yield of biopsy in our study is similar to prior studies, though the selected populations vary in the prior probability of osteomyelitis.^[6]^ Han and colleagues reported osteomyelitis in 14% of patients undergoing biopsy for stage IV decubitus pressure ulcers, but not all patients were suspected to have osteomyelitis.^[20]^ Wu et al reported a microbiologic yield of 34%, but only histologically proven cases of osteomyelitis were included in their analysis.^[6]^ Higher rates of culture-positivity have been demonstrated for patients who underwent surgery, perhaps because these represent more advanced disease.^[17,21–23]^ In our study, the only predictor of culture positivity was an elevated WBC count, which was not reported by others.^[6,24]^ Antibiotic therapy prior to biopsy similarly did not affect yield in a statistically significant way in our study.^[6]^ One retrospective study of non-vertebral osteomyelitis revealed a low diagnostic yield for IGB but was conducted when the procedure was less widespread, and the impact on antimicrobial therapy was not evaluated.^[6]^

Overall our histologic yield of IGB was somewhere between 29% and 78%. When accepting either positive histology or microbiology result as confirmatory of osteomyelitis, the sensitivity of IGB was 50%, similar to previous studies.^[10,25]^ Guidelines recommend analyzing bone samples for both microbiology and histology to improve diagnostic sensitivity.^[8,10]^ However, the utility of histology is controversial given the significant inter-observer variability in interpreting results.^[18,26,27]^ Its use may be limited in cases with high diagnostic certainty and in cases where patients are likely to receive antibiotics or surgery, regardless. While performing a biopsy may add value for a microbiologic diagnosis, splitting the biopsy sample may also reduce the volume of material sent for culture, thereby potentially decreasing the microbiology yield. Thus, the decision to send the material to pathology should hinge on whether the goal of the procedure is to confirm the diagnosis, or to obtain microbiologic information to help guide therapy.

IDSA guidelines recommend a 14-day antibiotic-free period prior to biopsy ^[8]^; however, we found that 79% of our patients had received antibiotics in the 30-days prior to biopsy, with a median antibiotic-free period of only 2 days. Clinicians may have been reluctant to withhold antibiotic therapy since the majority of patients presented with an overlying ulcer, cellulitis, and/or elevated inflammatory markers. We presume that the microbiologic yield might have been higher had the patients been off antibiotics for a longer period of time.

The results of IGB did not have a substantial impact on antibiotic choices in the majority of cases. Almost all patients who had been started empirically on antibiotics remained on antibiotics despite a negative culture, and more than half had no change to their regimen. Clinicians frequently initiated antibiotics despite a negative biopsy result. Empirically chosen regimens adequately treated bone organisms in all but three cases (1.5%), and final antibiotics, when prescribed, were still broader than expected from culture data alone in 93% of all cases.

Clinicians were more likely to withhold antibiotics in patients who had not received empiric therapy (65%) as compared to those who had (8%). While differences between these two subgroups contribute to this disparity, it also highlights the impact of cognitive biases on antibiotic prescribing behaviors. Physicians who initiated therapy for presumed osteomyelitis prior to obtaining biopsy results may be more prone to anchoring and framing effects than those who waited to make decisions.^[28]^ Ultimately, a closer analysis of providers’ thresholds for initiating or withholding treatment and covering key pathogens is needed to better understand the role of IGB cultures in antimicrobial choices.

This study includes the largest cohort of patients reported to-date with non-vertebral osteomyelitis and represents the heterogeneity of cases that clinicians encounter in practice. One limitation of the study is that not all patients included in the analysis had a definitive diagnosis of osteomyelitis, and physicians largely based their diagnosis on the clinical picture and supported by radiologic diagnosis. This may reflect an overreliance among clinicians on radiologic studies to diagnosis osteomyelitis. In the setting of negative biopsy results, clinicians tended to adhere to their presumptive diagnosis and prescribe long course of antibiotics.

Other limitations include the retrospective design, which weakened our ability to identify predictors of a positive biopsy due to inconsistencies in clinical documentation and in the availability of laboratory parameters. The data also comes from a single academic medical institution and may not adequately mirror the diversity of clinical practice. We were unable to discern the rationale behind clinicians’ choice of antibiotic management and cannot rule out the possibility that additional factors were considered in making changes, such as price, side effect profile or clinician preference. For instance, a broader spectrum agent, such as levofloxacin (which has anti-pseudomonal activity), may have been selected because of excellent oral bioavailability over narrower-spectrum agent, such as ceftriaxone, which requires infusion. Finally, we did not examine any short- or long-term clinical outcomes because of inconsistent availability of follow-up data.

The ideal indication for IGB is to confirm the diagnosis of osteomyelitis and to help tailor antimicrobial therapy to the offending organisms by getting more microbiologic data. Our study suggests that, in everyday clinical practice, for cases of non-vertebral osteomyelitis, patients are rarely off antibiotics for the recommended 14 days prior to biopsy, overall yield is low, and providers do not often tailor antimicrobial therapy based on obtained microbiologic data. IGB procedures increase the cost of care and frequently lead to prolonged hospitalizations, which further increases healthcare expenditure and risk of hospital-associated morbidity. Prospective studies looking at the role of imaging-guided bone biopsy in the management algorithm of non-vertebral osteomyelitis are needed to identify a subgroup of patients for whom this test would prove most useful.

## Acknowledgments

This work was accepted for poster presentation at Infectious Disease Week 2017, October 4–7, 2017 in San Diego, California. We are grateful to Dr. Navneet Narula (Department of Pathology, Weill Cornell) who helped with the interpretation of bone biopsy results and to Drs. Arthur Evans and Martin Shapiro for their critical reading of the manuscript. The authors have no potential conflicts of interest to disclose.

## Author contributions

**Conceptualization:** Cole B. Hirschfeld, Benjamin May, Ole Vielemeyer, Ernie L Esquivel.

**Data curation:** Cole B. Hirschfeld, Joanna Bryan, Deanna P. Jannat-Khah, Benjamin May.

**Formal analysis:** Cole B. Hirschfeld, Shashi N. Kapadia, Joanna Bryan, Deanna P. Jannat-Khah, Benjamin May, Ole Vielemeyer, Ernie L Esquivel.

**Funding acquisition:** Cole B. Hirschfeld, Ernie L Esquivel.

**Investigation:** Cole B. Hirschfeld, Shashi N. Kapadia, Ole Vielemeyer, Ernie L Esquivel.

**Methodology:** Cole B. Hirschfeld, Shashi N. Kapadia, Joanna Bryan, Deanna P. Jannat-Khah, Ole Vielemeyer, Ernie L Esquivel.

**Project administration:** Cole B. Hirschfeld, Ole Vielemeyer, Ernie L Esquivel.

**Software:** Joanna Bryan.

**Supervision:** Ole Vielemeyer, Ernie L Esquivel.

**Validation:** Ole Vielemeyer, Ernie L Esquivel.

**Writing – original draft:** Ole Vielemeyer, Ernie L Esquivel.

**Writing – review & editing:** Cole B. Hirschfeld, Shashi N. Kapadia, Joanna Bryan, Deanna P. Jannat-Khah, Benjamin May, Ole Vielemeyer, Ernie L Esquivel.

## Supplementary Material

Supplemental Digital Content
