# Supplementary material for: Impact of diagnostic bone biopsies on the management of non-vertebral osteomyelitis: A retrospective cohort study
Source: Medicine (Baltimore). 2019 Aug 23;98(34):e16954. doi: 10.1097/MD.0000000000016954 (PMC6716736; doi:10.1097/MD.0000000000016954)
Supplement: Supplemental Digital Content [file medi-98-e16954-s001.docx]

**Supplementary Table 1.** Definitions of antibiotic changes

| **Category** | **Definition** |
| --- | --- |
| Initiated | Patient without empiric antibiotics*, discharged with final antibiotics** |
| Discontinued | Patient treated with empiric antibiotics, discharged without final antibiotics |
| Broadened | Patient treated with empiric antibiotics, discharged with final antibiotics with addition of antimicrobial coverage against one or several of the following: MRSA, Pseudomonas, VRE and/or anaerobic organisms |
| Narrowed | Patient treated with empiric antibiotics, discharged with final antibiotics with removal of antimicrobial coverage against one or several of the following: MRSA, Pseudomonas, VRE and/or anaerobic organisms |
| Targeted | Patient treated with antibiotics specifically chosen to treat only organisms that were obtained from biopsy culture |
| No change | Patient discharged with final antibiotics, which were the same as empiric antibiotics. Also represents those who never received antibiotics post-biopsy and at discharge. |

**“Empiric antibiotics”* defined as antibiotics initiated after biopsy, prior to culture results being available.

***“Final antibiotics”* referred to antibiotics chosen after culture data from biopsy were available.

**Supplementary Table 2.** Microbiology results from wound culture

|  | N (%) |
| --- | --- |
| Number of samples | 60 |
| Microbiology-positive | 49 (81.7%) |
| Microbiology-negative | 11 (18.3%) |
| **Organisms** |  |
| Polymicrobial (> 2 organisms) | 29 (59.2%) |
| *Staphylococcus aureus* | 17 (34.7%) |
| Methicillin-sensitive | 12 (24.5%) |
| Methicillin-resistant (MRSA) | 5 (10.2%) |
| Coagulase-negative *Staphylococci* | 4 (8.2%) |
| *Streptococcus* spp. | 11 (22.4%) |
| *Enterococcus* spp., including VRE | 6 (12.2%) |
| *Coryneiform* spp | 13 (26.5%) |
| Lactobacillus | 1 (2.0%) |
| Enterobacteriaceae^δ^ | 17 (34.7%) |
| Pseudomonas aeruginosa | 5 (10.2%) |
| Non-enteric Gram-negative organisms^#^ | 3 (6.1%) |
| Anaerobes | 6 (12.2%) |

^δ^ Included Proteus, Escherichia, Enterobacter, Klebsiella, Serratia, Citrobacter, and Morganella species

^#^ Included Acinetobacter and Alcaligenes species.
